# Clinical application value of different SCC-Ag reference intervals

**DOI:** 10.1097/MD.0000000000042964

**Published:** 2025-06-27

**Authors:** Qi Xie, Longying Zhu, Liang Zhang, Shui Fu

**Affiliations:** a Department of Clinical Laboratory, First People’s Hospital of Linping District, Hangzhou, Hangzhou, Zhejiang Province, People’s Republic of China; b School of Medical Technology and Information Engineering, Zhejiang Chinese Medical University, Hangzhou, Zhejiang Province, China.

**Keywords:** diagnostic values, health checkup, positive rate, reference interval, SCC-Ag

## Abstract

This study aimed to establish a reference interval for squamous cell carcinoma antigen (SCC-Ag) that is tailored to a local population and to evaluate its clinical utility in comparison with the existing reference interval (≤1.5 μg/L) as recommended by the National Guide to Clinical Laboratory Procedures (4th Edition) and manufacturers. The study retrospectively analyzed data from 5251 healthy individuals to develop a locally applicable SCC-Ag reference interval, following guidelines from the CLSI-C28-A3c document and the WS/T 402-2012 Clinical Laboratory Test Project Reference Range. Subsequently, a cohort of 6191 healthy subjects was selected to evaluate the screening efficacy of the different SCC-Ag reference intervals. Additionally, 948 patients were included to assess the diagnostic performance of the 2 SCC-Ag reference intervals. The study proposes a reference interval for SCC-Ag of ≤2.2 μg/L. Utilizing this threshold, the positive rate among healthy subjects was 1.696%, significantly lower than the 7.931% rate based on the manufacturer-provided reference interval, indicating statistically significant differences. Furthermore, the sensitivity and negative predictive value of the newly proposed SCC-Ag reference interval were notably lower compared to the manufacturer’s reference interval. However, the specificity, positive predictive value, Youden index, and overall accuracy of the proposed interval exceeded those of the existing manufacturer-provided interval. Thus, in the context of screening healthy individuals and diagnosing patients, the SCC-Ag reference interval established in this study demonstrates superiority over the current manufacturer-provided interval. Consequently, this proposed reference interval may offer more effective information for clinical decision-making.

## 1. Introduction

The squamous cell carcinoma antigen (SCC-Ag) is a tumor-associated antigen that was first isolated from cervical squamous cell carcinoma by Kato and Torigoe in the 1970s.^[1]^ As a tumor marker, SCC-Ag has attracted much attention due to its biological function and significance in conventional physiological and pathological processes.^[2–4]^ SCC-Ag commonly occurs in minimal amounts in normal tissue and the epithelial cells of malignant lesions in different organs.^[5,6]^ As a squamous cell carcinoma (SCC) tumor marker with favorable specificity, SCC-Ag can be used in the diagnosis, curative effect monitoring, and prognosis evaluation of SCCs. Hence, it is also a reliable tumor marker for SCC diagnosis.^[7–9]^ The SCC-Ag reference interval is an important standard that allows clinicians to make diagnoses for patients, and its value may directly affect diagnostic accuracy.

Based on standard immunological principles, the chemiluminescent microparticle immunoassay (CMIA) system possesses high specificity. It can be employed to quantitatively detect SCC-Ag, avoiding the influence of jaundice, lipidemia, hemolysis, and other factors. The SCC-Ag level is related to tumor progression and can be used as an index for prognosis and recurrence.^[10–12]^ Currently, there are few authoritative reports on the application of CMIA systems in the measurement of SCC-Ag reference intervals in healthy individuals. The SCC-Ag reference interval provided by manufacturers and the *National Guide to Clinical Laboratory Procedures* (4th Edition)^[13]^ is ≤1.5 μg/L, which is a value adopted in most areas of China.

Currently, most CMIA systems for SCC-Ag detection in China are imported from foreign companies such as Roche, Abbott, Beckman, and Siemens. Gender, age, race, and region may affect SCC-Ag expression, but the guidelines for these systems all provide a reference interval based on individuals from other countries. This significantly impairs the reliability of the SCC-Ag reference interval in local clinical applications and may even lead to misdiagnoses or missed diagnoses. Although some researchers have investigated the SCC-Ag reference interval in various geographical areas, the sample sizes were small, and the SCC-Ag reference intervals were not compared with the reference interval that is commonly used in clinical practice (≤1.5 μg/L).

In recent years, routine tumor markers have become standard items in regular health checkups. As a conventional tumor marker, SCC-Ag is also included in the tumor screening of healthy individuals.^[14]^ Due to the large population of China, an inappropriate SCC-Ag reference interval may result in misdiagnoses and missed diagnoses for a considerable number of patients. Misdiagnoses result in ineffective medical examinations, which increase the economic burden on patients and may lead to additional mental stress. Besides, missed diagnoses may cause treatment delays and lead to further serious consequences.^[15]^ Hence, selecting an appropriate SCC-Ag reference interval maximizes the clinical application value of the SCC-Ag test. In this study, a local SCC-Ag reference interval was established based on authoritative guidelines. Moreover, the proposed SCC-Ag reference interval was compared with the one commonly used in clinical practice (≤1.5 μg/L). Identifying a more appropriate, high-efficiency local SCC-Ag reference interval lays a solid foundation for enhanced clinical diagnosis and treatment.

## 2. Materials and methods

### 2.1. Subjects and samples

**Figure 1.**
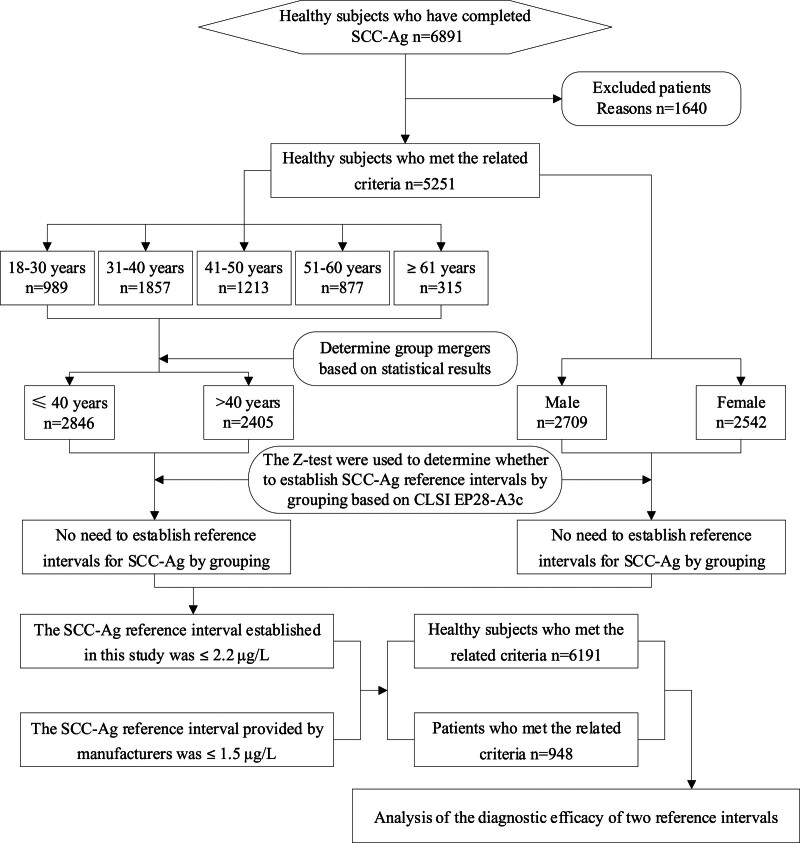
Design and flow of participants through the study.

This study is a retrospective analysis. In this study, healthy subjects who received health checkups at our hospital from January 2022 to June 2023 were selected as the SCC-Ag reference interval survey population. The study flow can be found in Fig. 1. They met the requirements of the Clinical and Laboratory Standards Institute (CLSI) EP28-A3c document^[16]^ and Appendix B “Reference Individual Survey Consultation Form” of the industry standard WS/T 402-2012 Clinical Laboratory Test Project Reference Range^[17]^ issued by the Ministry of Health of the People’s Republic of China. The inclusion criteria for the subjects were as follows: (1) aged over 18 years; (2) normal hepatic and renal function, normal cardiopulmonary function, and normal blood pressure, and no cardiovascular disease or history of tumors and other diseases; (3) no history of excessive alcohol consumption or smoking, no recent history of surgery or hospitalization, no obesity, diabetes, digestive tract diseases, hematological diseases, or hereditary diseases; (4) no abnormalities in other tumor indices. The exclusion criteria were: (1) a history of tumors; (2) autoimmune diseases or immunodeficiency diseases; (3) abnormal results from cervical liquid-based thin-layer cytology; (4) abnormal results from human papillomavirus tests; (5) abnormal chest computed tomography results, including inflammation, non-benign nodules, or tumors. There were 5251 eligible subjects, including 2709 males aged 18 to 90 years and 2542 females aged 19 to 81 years. Additionally, 6191 healthy individuals from July 2023 to December 2023 were also selected, including 2756 males aged 16 to 88 years and 3435 females aged 17 to 91 years. Moreover, 948 inpatients and outpatients with non-hepatitis, liver cirrhosis, pancreatitis, pneumonia, tuberculosis, respiratory insufficiency, renal insufficiency, psoriasis, and immune system diseases were selected, including 378 males aged 24 to 91 years and 570 females aged 17 to 92 years. These patients were diagnosed using at least 3 main routine examinations, including hepatic and renal function examinations, tumor marker examinations, and chest computed tomography; female patients also received human papillomavirus and Thinprep Cytologic Test examinations. According to the clinical diagnoses and examination results, these subjects were divided into positive and negative SCC-related index groups. Subsequently, the diagnostic efficacy was compared between the SCC-Ag reference interval established in this study and the SCC-Ag reference interval provided by manufacturers. This study was conducted with approval from the Ethics Committee of First People’s Hospital of Linping District, Hangzhou (No. 2021038). This study was conducted in accordance with the declaration of Helsinki. Written informed consent was obtained from all participants.

### 2.2. Instruments and consumables

SCC-Ag detection was performed using the Automated Immunoassay Analyzer (Alinity I, Abbott Laboratories, Chicago) and matching reagents (detection principle: chemiluminescent microparticle immunoassay), as well as standard products and quality control samples. These items demonstrated stable internal and favorable external quality assessment results, thereby guaranteeing accuracy.

### 2.3. Methods

The subjects followed a regular lifestyle and did not participate in any strenuous exercise for 3 days before SCC-Ag detection. After resting for 30 minutes, whole blood samples were collected in vacuum tubes without anticoagulant. The serum samples were kept at room temperature for 30 minutes and then centrifuged at 1200 g for 10 minutes. Next, 1 mL of serum from each sample was taken for detection (Abbott Alinity). All items were detected in strict accordance with the directions in the operating manuals.

### 2.4. SCC-Ag reference interval establishment

Firstly, we analyzed the SCC-Ag levels and evaluated the differences in SCC-Ag levels between different genders. The groupings were accomplished according to the statistical results. Following the CLSI EP28-A3c document and the WS/T 402-2012 *Clinical Laboratory Test Project Reference Range* issued by the Ministry of Health of the People’s Republic of China, we eliminated the outliers, determined whether the reference interval should be set in groups, and established the SCC-Ag reference interval.

### 2.5. Clinical application of different SCC-Ag reference intervals

The diagnostic efficacy and effectiveness of the SCC-Ag reference interval proposed in this study and the reference interval provided by manufacturers were measured and statistically analyzed based on the healthy screening population and respiratory patients.

### 2.6. Statistical analysis

SPSS 26.0 was used for data processing, and the Shapiro–Wilk normality test was employed to analyze the data distribution. The normally distributed measurement data were expressed as $\bar{x}\pm s$, and the *t* test was used for comparisons between groups. Data that were not normally distributed were expressed as M (Q1, Q3), and the rank sum test was used to compare non-normally distributed data between groups. The chi-square test was used for comparisons of rates, and *P* < .05 was considered to indicate statistical significance.

## 3. Results

### 3.1. Comparison of SCC-Ag between different genders and ages

**Figure 2.**
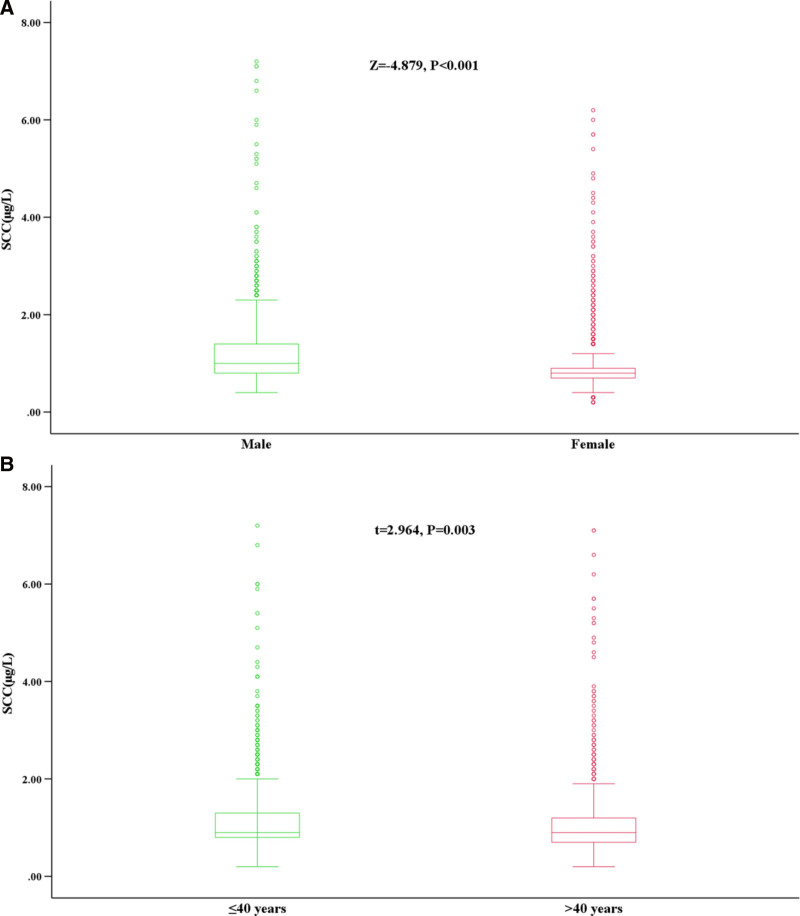
SCC-Ag levels of different gender and age groups. SCC-Ag = squamous cell carcinoma antigen (A) gender; (B) age.

Following the D/*R* ≥ 1/3 rule in the CLSI EP28-A3c document (D: the absolute difference between an extreme observation [large or small] and the next largest [or smallest] observation; R: the range of all observations), no outlier data were found in any of the 5251 subjects. Statistical analyses were conducted by grouping subjects according to different gender and age. There was a statistically significant difference in SCC-Ag levels between different genders (Table 1 and Fig. 2A). The comparison among different age groups showed that there were no significant differences in SCC-Ag levels between the 18 to 30 years and 31 to 40 years groups, nor among the 41 to 50 years, 51 to 60 years, and ≥61 years groups; however, there was a statistically significant difference between the 31 to 40 years and 41 to 50 years groups. Further analysis indicated that there were no statistical differences between any 2 groups among the 41 to 50 years, 51 to 60 years, and ≥61 years groups (Table 1). Based on the above statistical results, the age groups were merged into ≤40 years and >40 years groups. Further statistical analysis showed that there was also a significant difference between these 2 groups (Fig. 2B).

**Table 1:** Comparison of the SCC-Ag among different genders and age groups.

Item		Cases	SCC-Ag (μg/L)	*t* value	*P* value	*F* value	*P* value	Combining required
Gender	Male	2709	1.177 ± 0.624	13.261	*P* < .001	–	–	No
	Female	2542	0.995 ± 0.565					
Age(years)	18–30	989	1.095 ± 0.513	0.163*	.873	–	–	Yes
	31–40	1857	1.091 ± 0.612	3.098†	.002			
	41–50	1213	1.022 ± 0.597	‐1.366‡	.172	1.366	.255	Yes
	51–60	877	1.062 ± 0.627	‐0.188§	.823			
	≥61	315	1.070 ± 0.496	‐1.314∥	.189			

SCC-Ag = squamous cell carcinoma antigen.

* 18 to 30 years group compared with 31 to 40 years group.

† 31 to 40 years group compared with 41 to 50 years group.

‡ 41 to 50 years group compared with 51 to 60 years group.

§ 51 to 60 years group compared with ≥61 years group.

∥ 41 to 50 years group compared with ≥61 years group.

### 3.2. Grouping evaluation for establishing the SCC-Ag reference interval

The SCC-Ag reference interval for different genders and ages did not meet any of the 3 grouping conditions. Thus, it was not necessary to perform grouping for the SCC-Ag reference interval according to age or gender. Subsequently, an SCC-Ag reference interval was established according to the CLSI EP28-A3c document and the WS/T 402-2012 standard of the *Clinical Laboratory Test Project Reference Range* issued by the Ministry of Health of the People’s Republic of China (Table 2).

**Table 2:** Grouping evaluation results for establishing the SCC-Ag reference interval.

Group		Cases	$\bar{X}$ (μg/L)	s (μg/L)	Z	Z*	Grouping required
Gender	Male	2709	1.177	0.624	13.562	14.033	No
	Female	2542	0.955	0.565			
Age	≤40 years	2846	1.092	0.579	2.940	14.033	No
	>40 years	2405	1.043	0.636			

*Notes*: Z = $\frac{X1-X2}{\sqrt{\frac{s1^{2}}{n1}+\frac{s2^{2}}{n2}}}$, Z* = 3 $\sqrt{\frac{n1+n2}{240}}$. Grouping criteria: grouping is required if any of the following conditions are met: (1) s_2_ > 1.5 s_1_; (2) s_2_/(s_2_ − s_1_) < 3; (3) Z > Z*.^[16]^

SCC-Ag = squamous cell carcinoma antigen.

### 3.3. SCC-Ag reference intervals among different age groups

According to the CLSI EP28-A3c and WS/T 402-2012 standards, the decrease in SCC-Ag levels has little clinical significance. The upper limit of the reference interval was determined for the 5251 healthy subjects who met the inclusion criteria using the nonparametric method with unilateral P_95_. The SCC-Ag reference interval established in this study was ≤2.2 μg/L. The current SCC-Ag reference interval used by laboratories is provided in the manufacturer’s manual. This reference interval of ≤1.5 μg/L was verified using a small sample size and was conducted according to the WS/T 402-2012 standard (Table 3).

**Table 3:** SCC-Ag reference interval.

Item	Proposed reference interval		Reference interval provided by manufacturers (small number of reference subjects)	
	Cases (n)	Reference interval	Cases (n)	Reference interval
SCC-Ag (μg/L)	5251	≤2.2	20	≤1.5

SCC-Ag = squamous cell carcinoma antigen.

### 3.4. Application value of 2 SCC-Ag reference intervals in health check-ups

**Figure 3.**
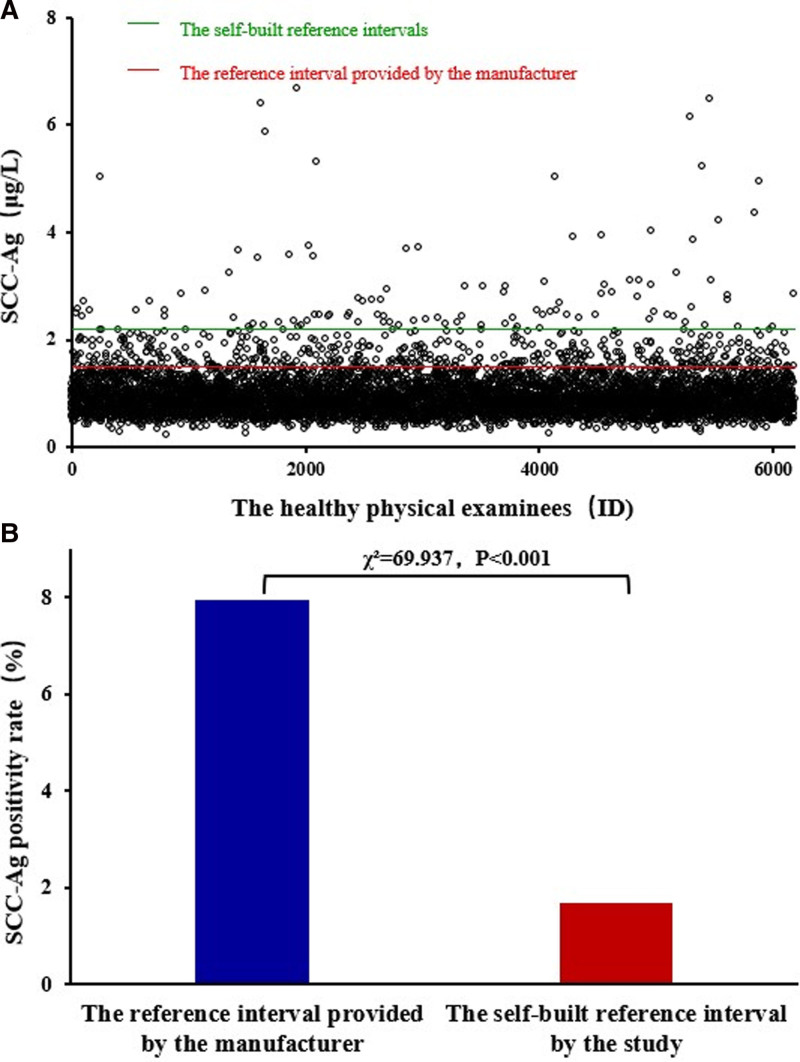
Positive SCC-Ag rate in healthy subjects. SCC-Ag = squamous cell carcinoma antigen (A) scatter plot of SCC-Ag in healthy examines; (B) SCC-Ag positive rates in healthy examines under different reference intervals.

Our proposed SCC-Ag reference interval and the one provided by manufacturers were used as diagnostic cutoff points in the health checkup center to calculate the positive rate among 6191 subjects. The positive rates based on the above 2 SCC-Ag reference intervals were 1.696% and 7.931%, respectively. Thus, the positive rate of healthy subjects based on our SCC-Ag reference interval was considerably lower than that of the SCC-Ag reference interval provided by manufacturers, and the difference was statistically significant (Fig. 3).

### 3.5. Diagnostic efficacy analysis of the 2 SCC-Ag reference intervals

The general information and disease types of 948 patients were classified and described. Then, the number of cases exceeding 2 SCC-Ag reference intervals was counted and summarized. The relevant results are shown in Table 4. The diagnostic efficacy of the 2 SCC-Ag reference intervals was assessed using the data from 948 patients. The sensitivity of the SCC-Ag reference interval established in this study was lower than that of the SCC-Ag reference interval provided by manufacturers. Moreover, the negative predictive value of our proposed SCC-Ag reference interval was slightly lower than that of the existing SCC-Ag reference interval. However, the specificity, positive predictive value, Youden index, and accuracy of the proposed SCC-Ag reference interval were all higher than those of the SCC-Ag reference interval provided by manufacturers (Table 5).

**Table 4:** General data of 948 patients with analyzed diagnostic value.

Item		Cases	Patients with squamous carcinoma			Patients with nonsquamous carcinoma		
			Case	SCC-Ag > 2.2 μg/L (cases/%)	SCC-Ag > 1.5 μg/L (cases/%)	Cases	SCC-Ag > 2.2 μg/L (cases/%)	SCC-Ag > 1.5 μg/L (cases/%)
Gender	Male	378	244	135/55.33	145/59.43	134	2/1.49	45/33.58
	Female	570	99	22/22.22	34/34.34	471	5/1.062	26/5.52
Age (years)	<45 years	347	10	8/80	10/100	337	3/0.89	10/2.97
	≥45 years	601	333	141/42.34	169/50.75	268	4/1.49	61/22.76
Type of disease	Respiratory diseases	307	258	119/46.12	137/53.10	49	0/0	16/32.65
	Cardiovascular disease	48	0	0/0	0/0	48	0/0	15/31.25
	Digestive disease	152	11	5/45.45	6/54.55	141	1/0.71	8/5.67
	Urological disease	34	0	0/0	0/0	34	1/2.94	14/41.18
	Gynecological disease	262	72	32/44.44	35/48.611	190	2/1.05	2/1.05
	Endocrine system disease	40	0	0	0/0	40	0/0	3/7.5
	Other diseases	105	2	1/50	1/50	103	3/2.91	13/12.62

*Notes*: All 258 patients with respiratory system squamous cell carcinoma had lung cancer, among whom 116 cases were at stage I, 105 cases at stage II, 27 cases at stage III, and 10 cases at stage IV; all 72 patients with gynecological system squamous cell carcinoma had cervical cancer, among whom 31 cases were at stage I, 20 cases at stage II, 16 cases at stage III, and 5 cases at stage IV. The clinical staging criteria for lung cancer and cervical cancer are referred to in references.^[18,19]^

SCC-Ag = squamous cell carcinoma antigen.

**Table 5:** Diagnostic value of different SCC-Ag reference intervals.

Reference interval	Sensitivity (%)	Specificity (%)	Positive predictive value (%)	Negative predictive value (%)	Youden index (%)	Accuracy (%)
Manufacturer-provided reference interval	52.19	88.26	71.60	76.50	40.45	75.21
Proposed reference interval	45.77	98.84	95.73	76.28	44.62	79.64

*Note*: Manufacturer-provided SCC-Ag reference interval is >1.5μg/L, proposed SCC-Ag reference interval is >2.2μg/L.

SCC-Ag = squamous cell carcinoma antigen.

## 4. Discussion

Based on statistical principles, this study conducted scientific grouping according to the standard methods specified in the CLSI EP28-A3c document and the industry standard WS/T 402-2012, “Establishment of Reference Intervals for Clinical Laboratory Tests,” issued by the National Health Commission, and established the reference interval for SCC-Ag in the healthy population of this region (≤2.2 μg/L). The SCC-Ag reference interval established in this study was higher than that reported Zhou et al, who set the upper limit at <1.70 μg/L. However, our reference interval was lower than that determined by Holdenrieder, who set a reference interval of <2.3 μg/L for a European population and <2.7 μg/L for a Chinese population.^[20,21]^ These inconsistencies in SCC-Ag may be related to variations in the geographic and physiological characteristics of the study populations, differences in sample size and selection criteria, disparities in detection methods and instruments, and differences in statistical analysis methods. The study subjects of Zhou Dongyi et al were healthy individuals from the Nanning area, while those of Holdenrieder et al included populations from Europe and China. These populations may differ from those in this study in terms of age, gender, race, and other factors, thereby affecting the natural variability of SCC-Ag levels. Additionally, differences in detection methods and techniques should not be overlooked. Different laboratories may have used different kits or platforms; even with the same technology, slight differences between different batches could lead to varying results. Finally, statistical processing methods and regional environmental factors such as dietary habits and lifestyle, also influence the determination of the final reference intervals.

This study established the SCC-Ag reference interval (≤2.2 μg/L) for the healthy population in the region by measuring serum SCC-Ag levels in 5251 healthy individuals undergoing physical examinations. Compared with the manufacturer’s recommended SCC-Ag reference interval (≤1.5 μg/L), the reference interval established in this study significantly reduced the positive rate among the healthy examinees (1.696% vs 7.931%). The diagnostic efficacy analysis showed that the SCC-Ag reference interval we established was superior to the manufacturer’s reference interval in terms of specificity, positive predictive value, Youden index, and accuracy. This indicates that using our reference interval can effectively exclude non-squamous cell carcinoma cases, helping to reduce false positives and offering certain advantages during diagnosis and monitoring stages. However, we also noted that the sensitivity of the reference interval established in this study is relatively low, and the negative predictive value has decreased. This means some true squamous cell carcinoma patients might be missed, which requires cautious consideration during the screening stage. Considering that SCC-Ag can also be elevated in various nonmalignant conditions, such as benign lung diseases, skin diseases like psoriasis, chronic kidney disease, etc,^[22,23]^ its use as a screening tool must be combined with other diagnostic methods. Therefore, balancing the relationship between sensitivity and specificity becomes particularly important.

In clinical practice, SCC-Ag plays a crucial role as a tumor marker in various applications, including screening, diagnosis, prognostic evaluation, treatment response monitoring, and recurrence monitoring.^[5]^ Different clinical applications may require distinct SCC-Ag reference intervals. During the screening phase, it is appropriate to select SCC-Ag reference intervals with higher sensitivity to minimize the risk of missing potential cases. However, this may increase the false-positive rate, leading to unnecessary treatment and psychological burden. In the processes of diagnosis and monitoring, specificity becomes more important to reduce the occurrence of misdiagnosis. To mitigate the negative impacts of misdiagnosis and missed diagnoses, multidisciplinary collaboration can be enhanced by integrating imaging examinations, histopathology, and other tumor markers to comprehensively assess the patient’s condition.^[1]^ Additionally, through education and training, the ability of medical personnel to interpret SCC-Ag test results can be improved, thereby avoiding misdiagnosis or missed diagnoses due to the limitations of SCC-Ag reference ranges. In the future, larger-scale clinical studies are needed to further validate and refine the reference ranges of SCC-Ag for different populations and disease states. Through evidence-based medicine, the most suitable SCC-Ag reference ranges for various clinical scenarios can be determined, ensuring that SCC-Ag maximizes its efficacy in clinical applications.

Currently, there are several reports concerning SCC-Ag reference intervals in different regions. These studies mostly focus on the establishment of the SCC-Ag reference interval. However, there are a limited number of studies that verify the SCC-Ag reference interval based on healthy subjects, and few in-depth studies assess the screening and diagnostic efficacy of the SCC-Ag reference interval. In this study, a local SCC-Ag reference interval was established, and its screening and diagnostic efficacy were comprehensively evaluated using health examination and patient data. The results revealed that our proposed SCC-Ag reference interval was superior to the SCC-Ag reference interval provided by manufacturers in terms of screening and diagnostic efficacy. The SCC-Ag reference interval established in this study demonstrates high diagnostic efficacy, facilitating clinical application and promoting the diagnosis and treatment of squamous cell carcinoma. Although this study achieved positive results, it still has certain limitations. Firstly, all data obtained from a single center, which may lead to biased results. Secondly, due to limitations in experimental conditions, not all factors that may affect SCC-Ag levels were included. Lastly, although we attempted to demonstrate the superiority of the new reference interval from multiple perspectives, long-term follow-up data are still limited. Future studies should consider multi-center collaboration, expanding sample coverage, and continuously tracking of participants’ health status to further verify the consistency and stability of the results. Additionally, exploring the potential of combining SCC-Ag with other biomarkers is a worthwhile research direction.

## 5. Conclusion

In this study, we established a China-specific SCC-Ag reference interval based on a large sample size, following authoritative guidelines from China and abroad (CLSI EP28-A3c and WS/T 402-2012 Clinical Laboratory Test Project Reference Range). In terms of screening effectiveness in healthy individuals and diagnostic efficacy in general patients, our proposed SCC-Ag reference interval was superior to the existing SCC-Ag reference interval commonly used in clinical practice. The current reference interval provided by manufacturers was verified with a small sample size and is based on the National Guide to Clinical Laboratory Procedures (4th Edition). Replacing the manufacturer-provided SCC-Ag reference interval with our proposed SCC-Ag reference interval will enable more effective decision-making in clinical practice.

## Author contributions

**Conceptualization:** Qi Xie, Longying Zhu, Liang Zhang, Shui Fu.

**Data curation:** Qi Xie, Longying Zhu, Liang Zhang.

**Formal analysis:** Qi Xie, Liang Zhang.

**Funding acquisition:** Shui Fu.

**Investigation:** Longying Zhu.

**Methodology:** Liang Zhang.

**Resources:** Liang Zhang.

**Software:** Longying Zhu.

**Supervision:** Shui Fu.

**Writing – original draft:** Qi Xie, Longying Zhu, Liang Zhang.

**Writing – review & editing:** Qi Xie, Longying Zhu, Liang Zhang, Shui Fu.
